# iSeeRNA: identification of long intergenic non-coding RNA transcripts from transcriptome sequencing data

**DOI:** 10.1186/1471-2164-14-S2-S7

**Published:** 2013-02-15

**Authors:** Kun Sun, Xiaona Chen, Peiyong Jiang, Xiaofeng Song, Huating Wang, Hao Sun

**Affiliations:** 1 Li Ka Shing Institute of Health Sciences, The Chinese University of Hong Kong, Shatin, New Territories, Hong Kong SAR, China; 2 Departments of Chemical Pathology, The Chinese University of Hong Kong, Shatin, New Territories, Hong Kong SAR, China; 3 Department of Obstetrics and Gynaecology, The Chinese University of Hong Kong, Shatin, New Territories, Hong Kong SAR, China; 4 Department of Biomedical Engineering, Nanjing University of Aeronautics and Astronautics, Nanjing 210016, China

## Abstract

**Background:**

Long intergenic non-coding RNAs (lincRNAs) are emerging as a novel class of non-coding RNAs and potent gene regulators. High-throughput RNA-sequencing combined with *de novo *assembly promises quantity discovery of novel transcripts. However, the identification of lincRNAs from thousands of assembled transcripts is still challenging due to the difficulties of separating them from protein coding transcripts (PCTs).

**Results:**

We have implemented iSeeRNA, a support vector machine (SVM)-based classifier for the identification of lincRNAs. iSeeRNA shows better performance compared to other software. A public available webserver for iSeeRNA is also provided for small size dataset.

**Conclusions:**

iSeeRNA demonstrates high prediction accuracy and runs several magnitudes faster than other similar programs. It can be integrated into the transcriptome data analysis pipelines or run as a web server, thus offering a valuable tool for lincRNA study.

## Background

Over the past decade, evidence from numerous high-throughput genomic platforms reveals that even though less than 2% of the mammalian genome encodes proteins, a significant fraction can be transcribed into different complex families of non-coding RNAs (ncRNAs) [1-4]. Other than microRNAs and other families of small non-coding RNAs, long non-coding RNAs (lncRNAs, >200nt) are emerging as potent regulators of gene expression [5]. Originally identified by Guttman et al. [6] from four mouse cell types using chromatin state maps as a subtype of lncRNAs, long intergenic non-coding RNAs (lincRNAs), are discrete transcriptional unit intervening known protein-coding loci. Recent studies demonstrate the functional significance of lincRNAs. However, it remains a daunting task to identify all the lincRNAs existent in various biological processes and systems.

Whole transcriptome sequencing, known as RNA-Seq, offers the promise of rapid comprehensive discovery of novel genes and transcripts [7]. With the *de novo *assembly software such as Cufflinks [8] and Scripture [6], a large set of novel assemblies can be obtained from RNA-Seq data. Several programs have been used to facilitate the cataloging of lincRNAs from RNA-Seq assemblies. For example, Li et al. [9] used Codon Substitution Frequency (CSF) score [10] to identify lincRNAs from *de novo *assembled transcripts in chicken skeletal muscle. Pauli et al. [11] took advantage of PhyloCSF score [12] followed by other filtering steps to identify lincRNAs expressed during zebrafish embryogenesis. Cabili et al. [13] also used PhyloCSF program to eliminate the *de novo *assembled transcripts with positive coding potential and identified ~8200 lincRNA loci in 24 human tissues. However, the extremely high computational times demanded by PhyloCSF, may become the bottleneck for handling millions of assemblies generated from high throughput sequencing. Furthermore, neither CSF nor PhyloCSF provides publicly available tools that can be readily integrated into the lincRNA identification workflow. Therefore, *ab initio *reconstruction of a reliable set of lincRNAs through computational method remains a daunting task. There is an urgent need for such a standalone tool to accurately and quickly distinguish lincRNAs from extremely large dataset. Previous studies showed that supervised machine learning method, especially Support Vector Machine (SVM), may represent a potential solution for accurate identification of lincRNAs and protein coding gene transcripts (PCTs). For example, CONC (Coding Or Non-Coding) [14], CPC (Coding Potential Calculator) [15], and POTRAIT [16] have been developed to discriminate PCTs and ncRNAs in general. However, the performance of these programs is largely dependent on datasets; for instance, CONC is slow on analyzing large datasets [15], which may limit its usefulness in the transcriptome data analysis. CPC works well with known PCTs but may tend to classify novel PCTs into lincRNAs if they have not been recorded in the protein databases used by CPC [15]. PORTAIT was specifically designed for the neglected species such as fungus et al. [16]. Moreover, their performance on the identification of lincRNAs has not been evaluated.

In this study, we present a new SVM-based classifier and a standalone tool, iSeeRNA. It demonstrated high accuracy, balanced sensitivity and specificity for both lincRNA and PCT datasets. It also outperforms others by running several order-of-magnitudes faster, thus representing an ideal tool for lincRNA identification from transcriptome sequencing data.

## Methods

### Standard input file formats

To be compatible with *de novo *assembly software, such as Cufflinks and Scripture, which use GTF/GFF or BED file format, we set these three formats as default input file formats for iSeeRNA. This will allow easy integration of iSeeRNA into the transcriptome data analysis workflow. The detailed information about the file formats can be found at UCSC genome browser (http://genome.ucsc.edu/FAQ/FAQformat.html).

### SVM settings

In order to build SVM models for iSeeRNA, we used LIBSVM (version 3.11) implementation [17] with Radial Basis Functional kernel which was shown to be the best kernel to deal with this task [15]. During the training, SVM was set as binary classifier with the two classes being lincRNAs (positive set) and PCTs (negative set). Optimized SVM parameters C and gamma were obtained by using the accompanying *grid.py *script with 5,000 randomly selected instances from the training dataset. To obtain the best performance model, 10-fold cross-validation was used. In addition, two models were trained and tested separately using species specific datasets for human and mouse, respectively.

### PhyloCSF and CPC settings

iSeeRNA was benchmarked against two other classification programs: PhyloCSF and CPC. These two programs were installed locally and executed with default parameters. For PhyloCSF, a score of 0 was used as the classification parameter. For CPC, Uniref90 [18] was employed as protein database and the default classification model developed by its authors was used.

### Performance measurements

To evaluate the performance, accuracy (sensitivity or specificity) and Matthews Correlation Coefficient (MCC) [19], an indicator used in machine learning as a measure of the quality of binary (two-class) classification, were calculated; and Receiver Operating Characteristic (ROC) curves were generated.

The following equations were used for calculating sensitivity and specificity:

(1) $$Sensitivity=\frac{TP}{TP+FN}$$

(2) $$Specificity=\frac{TN}{TN+FP}$$

(3) $$MCC=\frac{TP^{*}TN-FP^{*}FN}{\sqrt{\left(TP+FP\right)\left(TP+FN\right)\left(TN+FP\right)\left(TN+FN\right)}}$$

Where TP, FP, TN and FN are the numbers of true positives (lincRNAs predicted to be non-coding), false positives (PCTs predicted to be non-coding), true negatives (PCTs predicted to be coding) and false negatives (lincRNAs predicted to be coding).

## Results

### Gold-standard datasets

**Figure 1:**
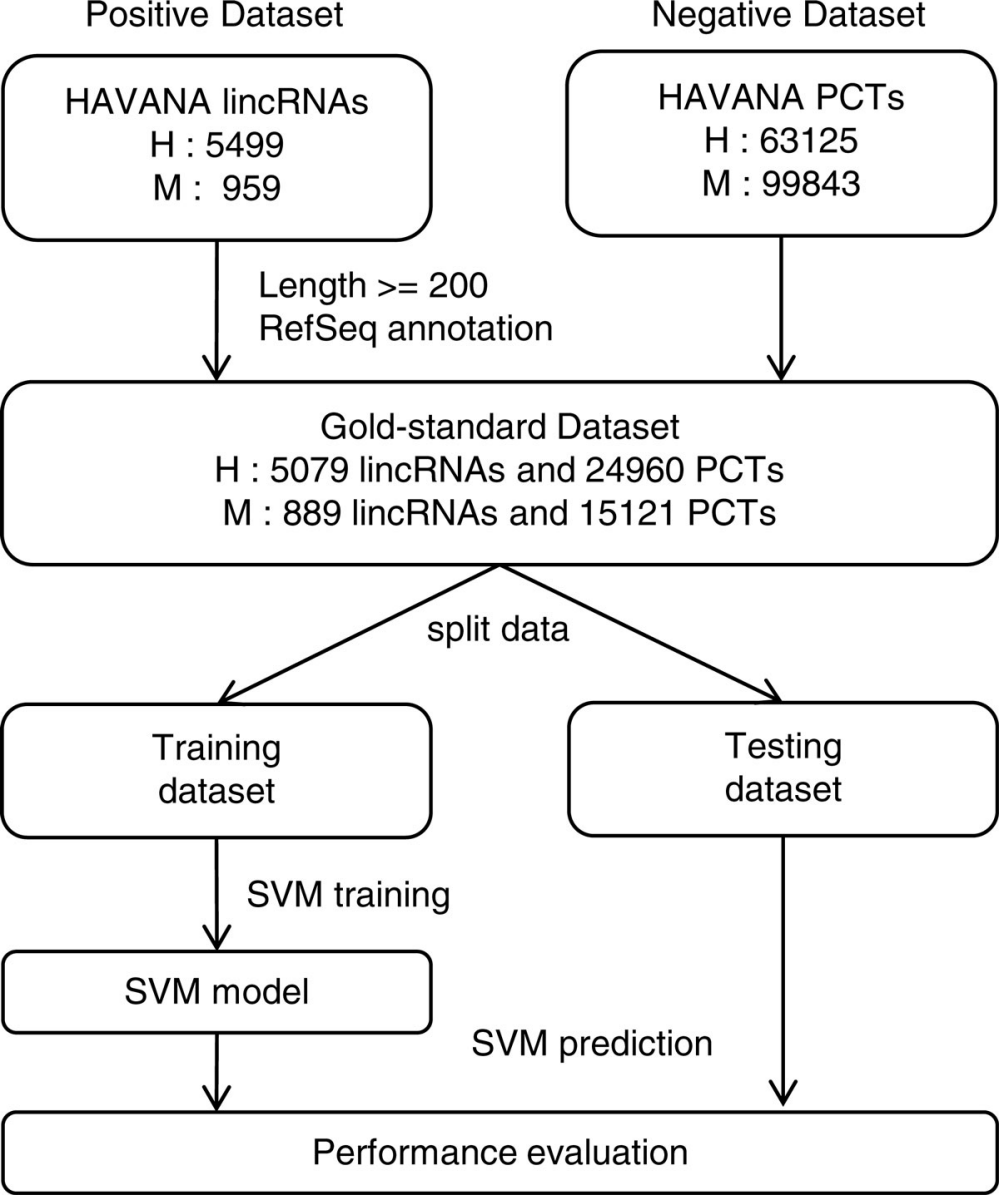
**Datasets and workflow of iSeeRNA**. Annotated lincRNAs (positive dataset) and PCTs (negative dataset) were collected from HAVANA project (H, human; M, mouse). After filtering through transcript length and RefSeq annotations, Gold-standard datasets were obtained and further split into training and testing datasets, which were then used for performance evaluation.

The quality of the training data is ultra-important for building an accurate SVM model. In order to obtain a pool of high quality lincRNAs and PCTs as Gold-standard datasets (Figure 1), we collected lincRNAs and PCTs annotated either as "known" or "novel" from Human and Vertebrate Analysis and Annotation (HAVANA) (http://vega.sanger.ac.uk/index.html) [20] project. These lincRNA annotations were manually curated and supported by some experimental evidences such as spliced cDNAs and ESTs et al., thus providing an ideal source for lincRNAs. We further filtered the data with the transcript length (> 200 nt). Next, for lincRNAs, we eliminated those transcripts that were annotated as PCTs by RefSeq [21]; similarly, for PCTs, we only kept those transcripts that have consistent annotations in both HAVANA and RefSeq. As a result, we created a Gold-standard dataset with a total of 30,039 transcripts for human, including 5,079 lincRNAs and 24,960 PCTs. A total of 16,010 transcripts were collected for mouse, including 889 lincRNAs and 15,121 PCTs. In order to generate the training and testing datasets from this Gold-standard dataset, we randomly selected half of the lincRNAs and roughly equal number of PCTs to form a balanced training dataset. We therefore obtained a training dataset with 2,594 lincRNAs and 2,583 PCTs for human, and a second one with 424 lincRNAs and 465 PCTs for mouse (Figure 1). The remaining lincRNAs and PCTs in Gold-standard dataset were combined to form two testing datasets for human and mouse separately.

### Feature selection

Selecting appropriate features is one of the most critical steps to build a SVM classifier. Many features have been used in distinguishing ncRNAs from PCTs. These can be classified to three categories: conservation, Open Reading Frame (ORF) and nucleotide sequences-based [12,14-16,22-25]. We employed those features that have demonstrated good potential to differentiate PCTs from ncRNAs in general considering lincRNAs share some common sequence properties with other classes of ncRNAs. As a result, a total of 10 features in three categories were used to build our SVM models. The first class of feature is conservation. Many studies have demonstrated that lincRNAs are less conserved than PCTs in general [13], making this a suitable feature for distinguishing them. To calculate the conservation score, we first downloaded the base-resolution phastCons [26] score files from UCSC; the scores of all nucleotides were then collected and averaged to obtain the conservation score for each transcript. The homolog search based features were among the most popular features for ncRNA classification but not employed for the following reasons. First, many novel PCTs are not collected in the protein database so that they tend to be mis-classifed as ncRNAs; Second, it showed strong correlation with the conservation score (Spearman correlation = 0.728, see Additional file 1), which did not further improve the performance when conservation is used. Lastly, it is very demanding in terms of computational time so that it tremendously reduces the performance of SVM classifier. Two Open Reading Frame (ORF) related features were selected as the second class, i.e. ORF length and ORF proportion defined by the length of an ORF divided by the total length of the transcript. We reasoned that a true lincRNA transcript, compared to PCTs, is more likely to have a low-quality ORF reflected by either a short ORF or a small proportion. txCdsPredict program from UCSC genome browser was employed to calculate the ORF for each transcript; the other seven features constitute the third class including frequencies of seven di- or tri-nucleotide sequences (GC, CT, TAG, TGT, ACG and TCG), which contribute the most to the overall performance. Some other nucleotide based features were not employed due to their weak classification ability [16]. We found that all the three classes were useful to some extent in distinguishing lincRNAs and PCTs when used alone; and exon conservation score and ORF proportion showed the highest discrimination power among all the features. (see Additional File 2).

### Performance evaluation

**Figure 2:**
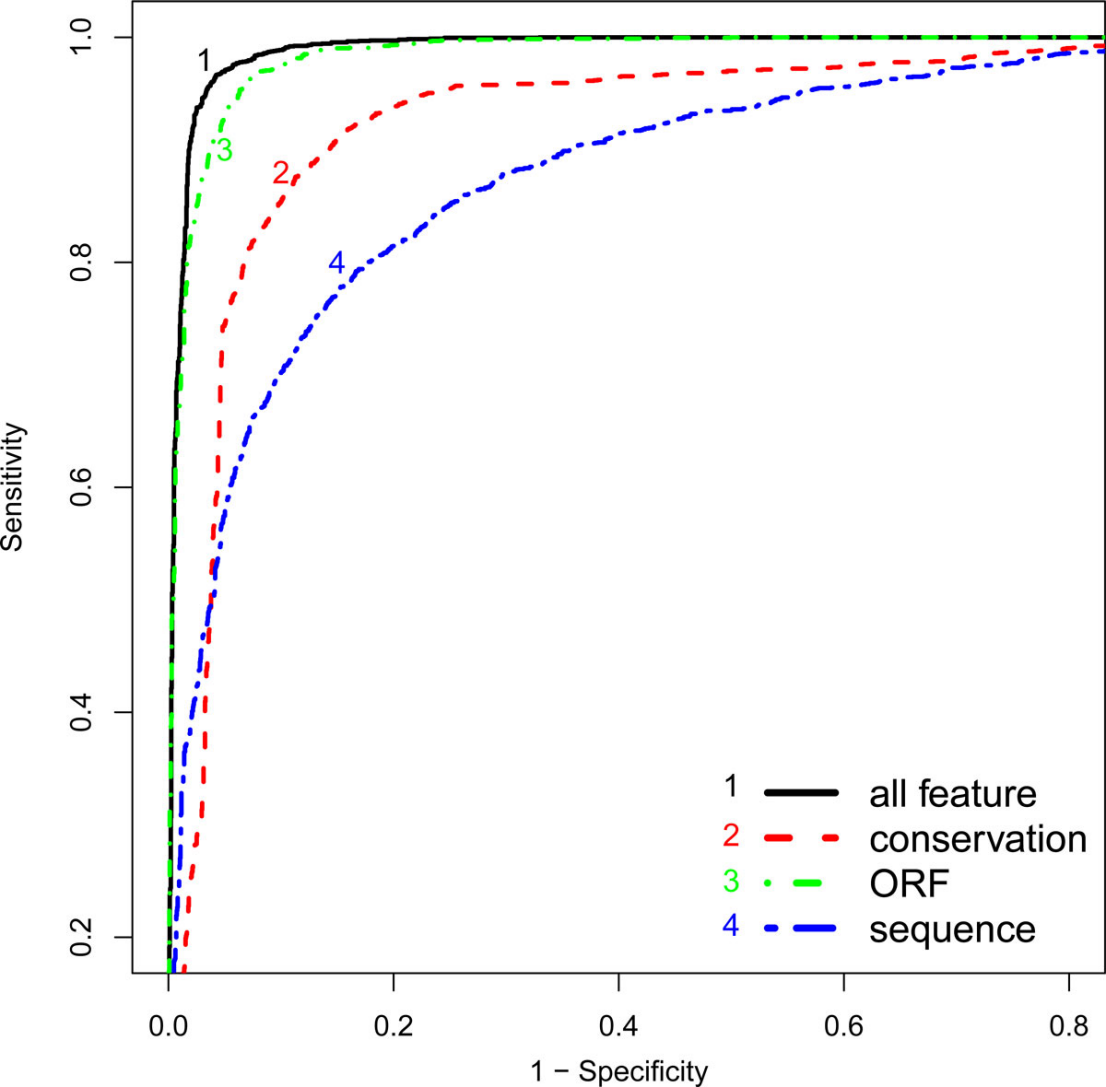
**The ROC curves for SVMs trained with different feature classes**. The true positive rate (i.e., sensitivity) was plotted against the false positive rate (i.e., 1-specificity). (1). All three classes of features combined. (2). ORF only; (3). Conservation only; (4). Nucleotide sequence based features only.

Using ROC, we first evaluated the performance of iSeeRNA when using three classes of features independently or in combination. As shown in Figure 2, each class is capable of distinguishing but the combination of all features led to the best performance. This justified the need of using all 10 features for building the trained SVM models for iSeeRNA. During the training, iSeeRNA presented a 10 cross-validation accuracy of 95.4% and 94.2% on training datasets of human and mouse respectively. When applied the trained models on the testing datasets, iSeeRNA showed an accuracy of 96.1% (2,387/2,485) on lincRNAs and 94.7% (21,200/22,377) PCTs for human testing dataset (Table 1). Similarly, iSeeRNA correctly predicted 94.2% (438/465) lincRNAs and 92.7% (13,632/14,702) PCTs for mouse testing dataset (Table 1).

**Table 1:** Performance evaluation of iSeeRNA on testing datasets

Species	Dataset	Data size	Prediction	Accuracy(%)
Homo Sapiens	lincRNAs	2485	2387	96.1
	PCTs	22377	21200	94.7

Mus Musculus	lincRNAs	465	438	94.2
	PCTs	14702	13632	92.7

We further evaluated iSeeRNA performance on several benchmark datasets collected from published studies. The first dataset is a collection of experimentally validated functional lincRNAs (28 for human and 11 for mouse). iSeeRNA successfully identified these transcripts as lincRNAs with 100% accuracy. We then applied iSeeRNA on a collection of 8,195 human lincRNAs identified from *de novo *assembled transcripts [13], iSeeRNA correctly predicted 97.3% (7,977/8,195) of these lincRNAs (data not shown). These results further demonstrated the high accuracy of iSeeRNA for the identification of lincRNAs.

### Comparison to other methods

**Figure 3:**
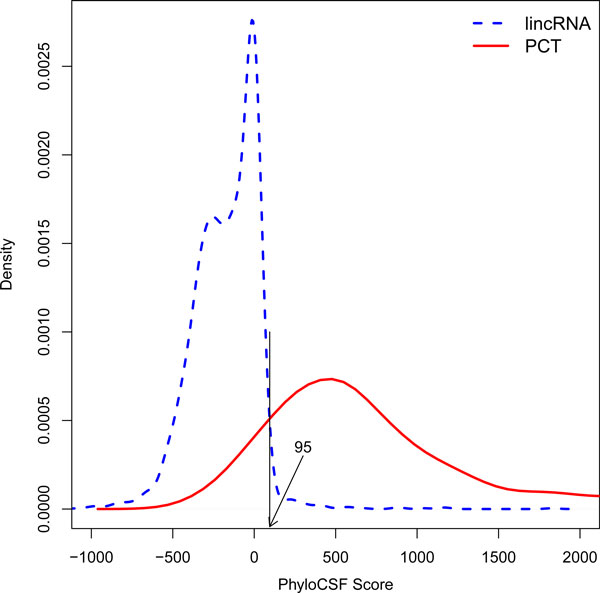
**Distribution of PhyloCSF score**. Distribution of PhyloCSF score for lincRNAs (dotted line) and PCTs (solid line). The overlapping point of the two density lines (shown by the vertical line) indicates the optimal PhyloCSF score for lincRNA identification is 95 (arrow).

Next we compared iSeeRNA performance with PhyloCSF and CPC. Since the number of well-annotated PCTs is much higher than that of lincRNAs, in order to have a fair comparison, we created a balanced comparison dataset from the Gold-standard dataset collected before (Figure 1). This dataset includes all 2,485 lincRNAs and 2,432 PCTs selected from the human testing dataset which did not appear in the training dataset. When iSeeRNA, CPC*, and *PhyloCSF were applied on this dataset, at the default thresholds, iSeeRNA demonstrated the best overall performance measured by MCC (0.935) followed by CPC (0.854) and PhyloCSF (0.770). iSeeRNA also showed the highest specificity (95.3%) (Table 2). Additionally, iSeeRNA displayed a better sensitivity (96.1%) compared to PhyloCSF (82.9%), but lower than CPC (99.2%) (Table 2). We have to point out that PhyloCSF failed to give scores for 34 (1.37%) lincRNAs in the comparison dataset; thus the calculation of the prediction accuracy and CPU time for PhyloCSF was based on the remaining 2,452 lincRNAs (Table 2). In addition, we plotted the distribution of the PhyloCSF scores (Figure 3) and found that the optimal cutoff to achieve the best performance was 95 instead of 0 as in default. At this cutoff, PhyloCSF displayed a high sensitivity of 97.9% but the specificity was dramatically reduced to 87.1%.

**Table 2:** Evaluation of accuracy and CPU time of iSeeRNA, PhyloCSF, and CPC on comparison dataset

Dataset	Data size	Accuracy (%)			Running Time^c^		
		
		iSeeRNA		CPC^b^	iSeeRNA	PhyloCSF^d^	CPC
lincRNAs	2485	96.1	82.9	99.2	19.2s	3270m	278s

PCTs	2432	95.3	92.0	85.2	25.7s	13307m	309s

^a ^PhyloCSF failed to calculate the score for 34 lincRNAs; the accuracy and CPU time were thus calculated based on 2451 lincRNAs.

^b ^Uniref90 was used as the protein database.

^c ^m, minute; s, second.

^d ^To save the running time, we split the comparison dataset and ran PhyloCSF on 20 nodes in parallel. The reported CPU time was the sum of the execution time on all nodes.

**Figure 4:**
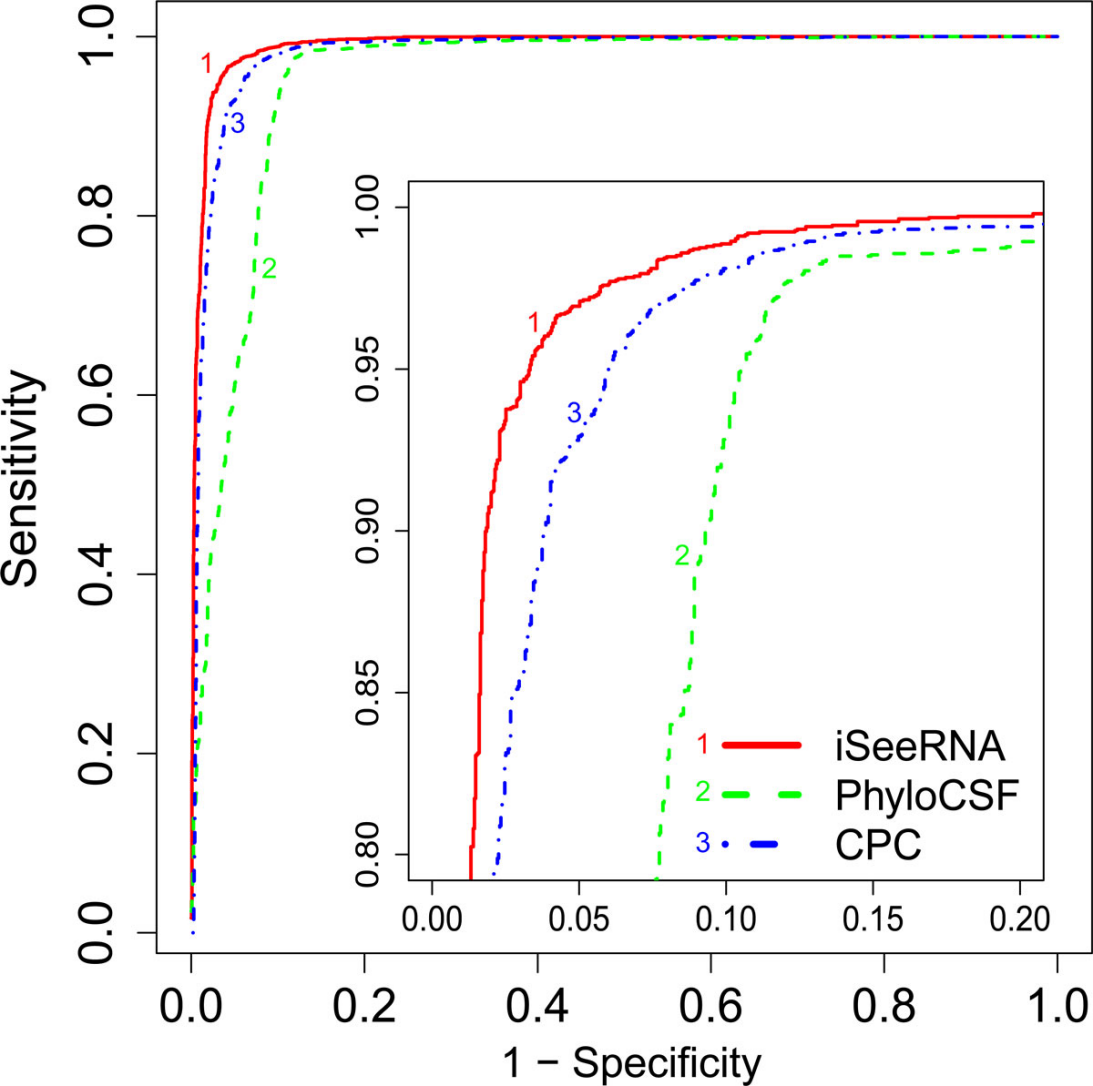
**ROC Comparison**. ROC curves of iSeeRNA (1), CPC (2) and PhyloCSF (3). Sensitivity was plotted against (1 - specificity), allowing comparison of three classifiers. The small figure inside displays the enlargement of the top-left corner.

To strengthen the above findings, we generated ROC curves (Figure 4) and calculated the Area Under the Curve (AUC) which measures the overall performance of a method under different thresholds. The AUC of iSeeRNA is above 0.99 indicating an excellent classifier. Compared to the AUC of CPC (0.98) and PhyloCSF (0.95), iSeeRNA further demonstrated the best overall performance with balanced sensitivity and specificity.

To test the efficiency of iSeeRNA, we next recorded the computational times for these three methods on the comparison dataset. Overall, iSeeRNA showed several order-of-magnitudes faster than PhyloCSF and at least 10 times faster than CPC (Table 2). This suggests that iSeeRNA is more suitable for processing large amount of transcripts from high-throughput transcriptome sequencing data. This advantage together with accepting GFF/GTF/BED as input file format makes iSeeRNA an ideal program that can be smoothly integrated as part of a lincRNA annotation pipeline for high-throughput transcriptome data analysis.

### Web implementation

**Figure 5:**
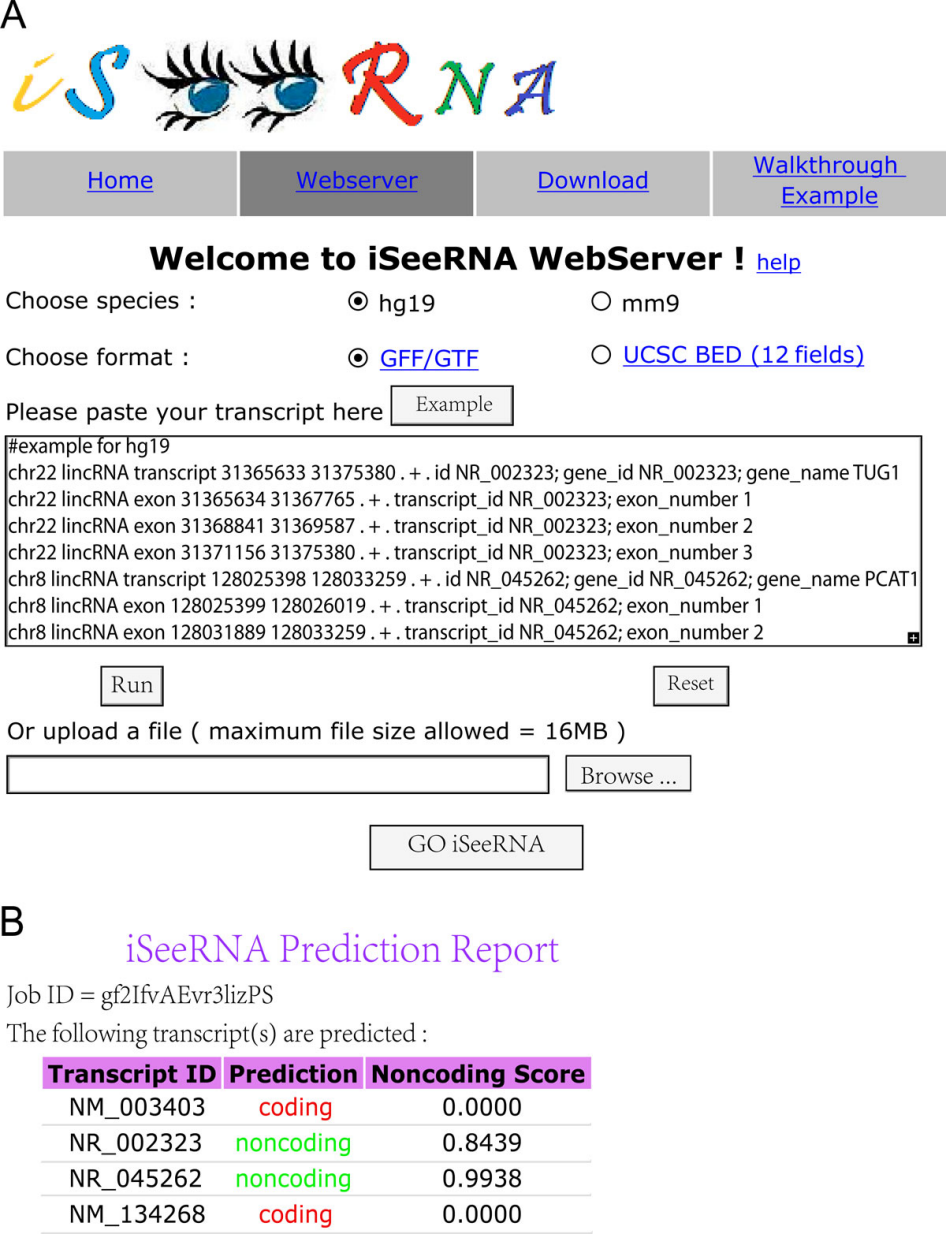
**Screenshots of iSeeRNA web server**. (A). Job submission page. An example dataset in GTF format is used as input file. (B). iSeeRNA output page. iSeeRNA reports transcript ID, prediction result, and a noncoding score.

Next, to facilitate the use of iSeeRNA, we implemented it as a user-friendly web server with free accessibility at http://www.myogenesisdb.org/iSeeRNA (Figure 4). The current web server provides trained SVM models for two species, human and mouse. The input file of the iSeeRNA web server can be in GFF/GTF or BED format. Users can either input their data into the text area of the web server or upload their input file (Figure 5A). The web server can process thousands of transcripts simultaneously. The outputs include sequence ID, predicted category and a non-coding score (Figure 5B); the score is highly correlated with prediction accuracy (see Additional file 3), for example, a 0.95 iSeeRNA non-coding score corresponds to approximately 95% possibility of the transcript to be non-coding.

## Discussion

In this study, we report a lightweight SVM-based program, iSeeRNA, designed for computational identification of lincRNAs from high-throughput transcriptome sequencing data. We have provided not only a standalone program that can be integrated into the transcriptome data analysis pipeline but also a web server for those with limited bioinformatics support to use it independently. Compared to similar programs, iSeeRNA directly support the file formats widely used by the RNA-Seq assemblers, and it also has demonstrated the best performance in terms of the prediction accuracy for both lincRNAs and PCTs and the computational time. We think this stems from the following improvements we have made in terms of feature selection, training dataset used and optimization of the computational method: (i) iSeeRNA was uniquely trained in a species-dependent manner. By using species-specific lincRNA and PCT training datasets, we have built two separate SVM models for human and mouse respectively. However, iSeeRNA also allows users to build additional customized models for the species of their interest with the increasing number of species-specific lincRNAs discovered at a rapid speed; (ii) iSeeRNA was trained with a balanced dataset containing approximately equal number of lincRNAs and PCTs. This has avoided the overfeeding of protein coding data and potential bias during the performance evaluation thus leading to accurate prediction with a balanced sensitivity and specificity. (iii) Compared to CPC, iSeeRNA does not use any homolog based features (such as the BLASTX [27] score) derived from homolog search results. As novel PCTs are likely omitted in the database, these features showed bias towards lincRNAs which may explain why CPC achieved a higher sensitivity but a comparatively lower specificity (Table 2). In addition, iSeeRNA employed seven sequence based features which were not considered by CPC. (iv) Unlike PhyloCSF, which is solely based on conservation for evaluating the coding potential of a transcript, iSeeRNA integrates multiple features. Our results demonstrated that PhyloCSF had difficulty in making clear discrimination between lincRNAs and PCTs. Even at the optimal threshold (95), 12.9% PCTs were wrongly classified as lincRNAs (Figure 3). However, the classification performance was clearly improved by integrating more features in iSeeRNA (Figure 2). Furthermore, PhyloCSF failed to calculate the scores for some of the HAVANA annotated lincRNA transcripts (Table 2), this further limits its application on lincRNA identification.

## Conclusions

In conclusion, we have implemented a highly accurate and reliable tool, iSeeRNA, for high throughput screening of lincRNAs from transcriptome sequencing data. We provided not only a web server for small dataset but also a standalone program that can be integrated into a bioinformatics pipeline for complex transcriptome data analysis. iSeeRNA demonstrates high performance with high accuracy and balanced sensitivity and specificity for both lincRNAs and PCTs. This makes it a valuable tool for lincRNA studies.

## Competing interests

The authors declare that they have no competing interests.

## Authors' contributions

KS, HW and HS conceived the study, designed and implemented the software; XC, PJ and XS participated in software design and provided technical assistance. KS, HW and HS wrote the manuscript. All authors read and approved the final manuscript.

## Supplementary Material

Additional file 1 **Details of conservation score and the blastx score for the comparison dataset**. Click here for file

Additional file 2 **Comparison of the potential for each feature on the discrimination of lincRNAs (red) from PCTs (green)**. The calculated feature values were normalized to values between 0 and 1. Each feature can distinguish lincRNAs from PCTs to some extension. Exon cons (exon conservation score) and ORF proportion shows the highest discrimination power among all the features. Click here for file

Additional file 3 **Scatter plot of iSeeRNA the prediction accuracy and noncoding score**. Click here for file

## Declarations

The publication costs for this article were funded by the University Grants Committee of the Government of the Hong Kong Special Administrative Region, China, under the General Research Funds (CUHK473211).

This article has been published as part of *BMC Genomics *Volume 14 Supplement 2, 2013: Selected articles from ISCB-Asia 2012. The full contents of the supplement are available online at http://www.biomedcentral.com/bmcgenomics/supplements/14/S2.
