# Supplementary figures and images for: iSeeRNA: identification of long intergenic non-coding RNA transcripts from transcriptome sequencing data
Source: BMC Genomics. 2013 Feb 15;14(Suppl 2):S7. doi: 10.1186/1471-2164-14-S2-S7 (PMC3582448; doi:10.1186/1471-2164-14-S2-S7)

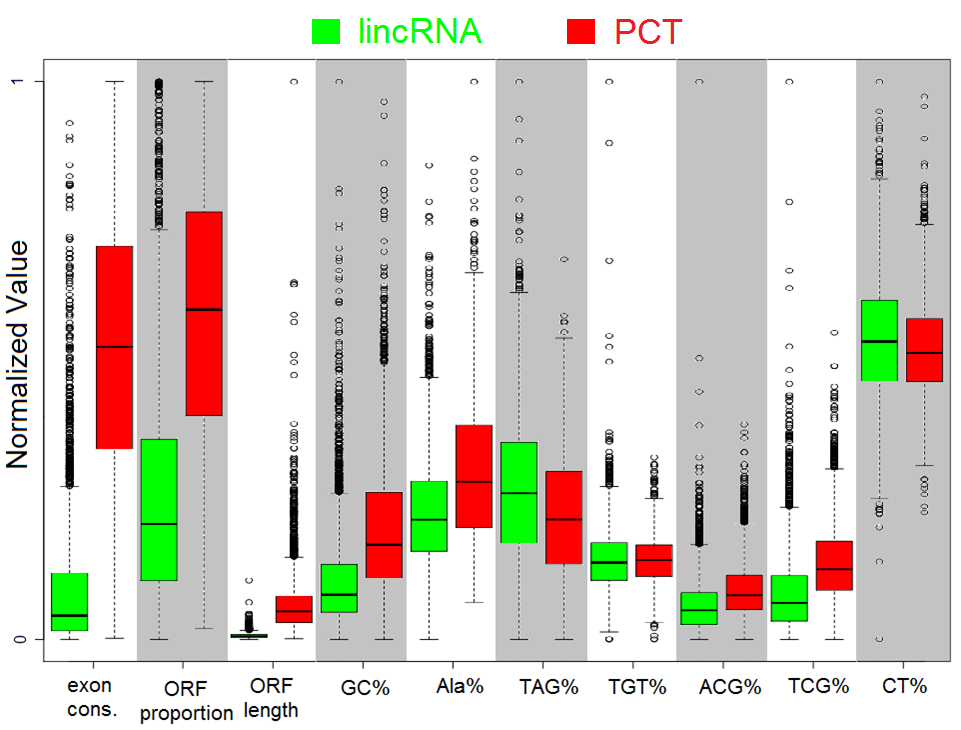

Supplement: Additional file 2 — Comparison of the potential for each feature on the discrimination of lincRNAs (red) from PCTs (green). The calculated feature values were normalized to values between 0 and 1. Each feature can distinguish lincRNAs from PCTs to some extension. Exon cons (exon conservation score) and ORF proportion shows the highest discrimination power among all the features. [file 1471-2164-14-S2-S7-S2.png]

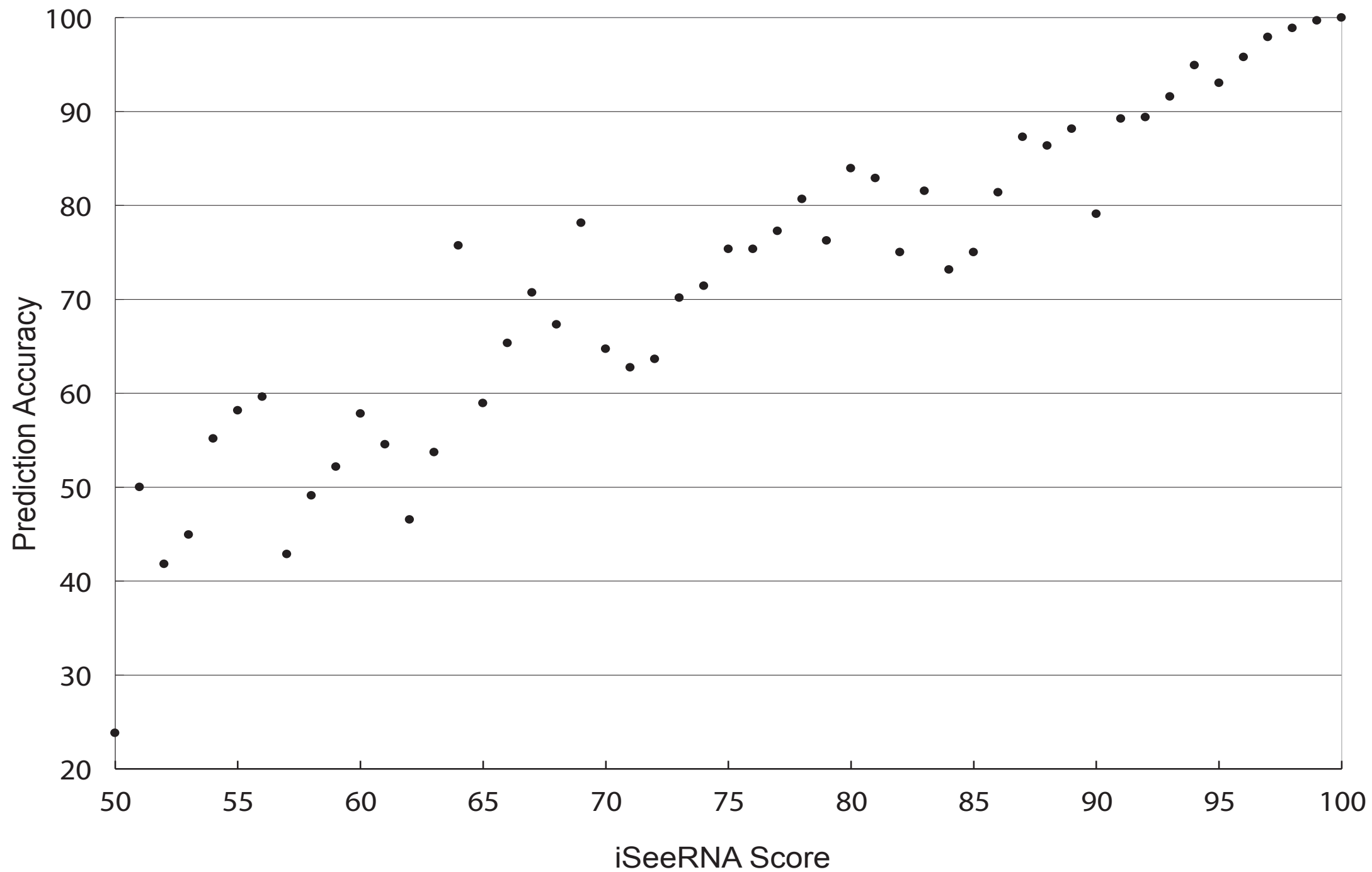

Supplement: Additional file 3 — Scatter plot of iSeeRNA the prediction accuracy and noncoding score. [file 1471-2164-14-S2-S7-S3.pdf]
